# Feasibility and Acceptability of Screening and Brief Interventions to Address Alcohol and Other Drug Use among Patients Presenting for Emergency Services in Cape Town, South Africa

**DOI:** 10.1155/2012/569153

**Published:** 2012-11-06

**Authors:** Bronwyn Myers, Dan J. Stein, Bulelwa Mtukushe, Katherine Sorsdahl

**Affiliations:** ^1^Alcohol and Drug Abuse Research Unit, South African Medical Research Council, Francie Van Zijl Drive, 7505 Parow, South Africa; ^2^Department of Psychiatry and Mental Health, Faculty of Health Sciences, University of Cape Town, 7001 Cape Town, South Africa

## Abstract

Despite evidence from high income countries, it is not known whether screening and brief interventions (SBI) for alcohol and other drug (AOD) use are feasible to implement in low and middle income countries. This paper describes the feasibility and acceptability of a peer-led SBI for AOD-using patients presenting with injuries at emergency services in Cape Town, South Africa. Data were extracted from program records on the number of eligible patients screened and the number of program refusals. A questionnaire examined preliminary responses to the intervention for 30 patients who had completed the program and 10 emergency personnel. Peer counselors were also interviewed to identify barriers to implementation. Of the 1458 patients screened, 21% (305) met inclusion criteria, of which 74% (225) were enrolled in the intervention. Of the 30 patients interviewed, most (83%) found the program useful. Emergency personnel were supportive of the program but felt that visibility and reach could improve. Peer counselors identified the need for better integration of the program into emergency services and for additional training and support. In conclusion, with limited additional resources, peer-led SBIs for AOD use are feasible to conduct in South African emergency services and are acceptable to patients and emergency personnel.

## 1. Introduction

South Africa has high rates of alcohol and other drug (AOD)-related problems, with these problems being particularly prevalent in the Western Cape Province of the country. For example, findings from a recent nationally representative survey indicate that the lifetime prevalence for any AOD use disorder (defined by DSM-IV criteria for abuse or dependence) was 20.3% in this province, which far exceeds the national average of 13.3% [1]. Furthermore, according to several nationally representative surveys, the Western Cape has one of the highest prevalence rates of hazardous and harmful alcohol use in the country [2, 3]. 

These high levels of problematic AOD use are a major concern for public health in the province, especially given evidence that AOD use is strongly associated with interpersonal violence and injury [4–8], which is the second leading cause of life years lost in the province after HIV/AIDS [9]. Evidence from earlier studies suggests that AOD use is associated with interpersonal violence and injury in several ways. First, AOD use leads to disinhibition which can trigger aggressive behavior and violence [10–12]. Second, people who are intoxicated are more likely to become victims of violence; mainly because AOD use impairs cognition and decision making which may impact one's ability to identify and avoid potentially dangerous situations [10, 13, 14]. In addition, victims of violence and injury have an increased likelihood of using AODs to cope with the experience of victimization and injury [9, 13–16]. 

This association between AOD use and risk for violence and injury suggests that screening and brief interventions (SBI) to reduce AOD use may be useful for preventing AOD-related violence and injury in the province [17]. As close to half of the individuals presenting with injuries at emergency rooms in the province have been using AODs [6–8], these settings are potentially good locations for identifying individuals (through screening) at risk for AOD-related problems who would not normally seek treatment and for conducting brief interventions to reduce their AOD use and risk for future AOD-related injuries. 

However, there are several limitations to current knowledge on SBI for AOD use that need to be considered prior to implementing these interventions in South African emergency rooms. First, while there is substantial evidence that SBI is effective for reducing problematic alcohol use among patients attending general health care services [18–20], corresponding research on the effectiveness of SBI for illicit drug use is sparse [21]. Although a handful of studies from high income countries report that SBI results in significant short-term reductions in illicit drug use [22–25], the body of evidence is still too small to make definitive statements about their effectiveness. Second, most of the studies showing that SBI is effective for reducing alcohol and illicit drug use were situated in primary health care settings and evidence for the effectiveness of SBI when delivered in emergency departments is equivocal [26–28]. For example, although nine of the 14 studies included in a systematic review of BIs conducted among patients presenting with injuries in emergency rooms demonstrated positive reductions in alcohol consumption, five of the 14 studies did not find significant differences in alcohol intake across the compared conditions [26]. In addition, a meta-analysis of 13 studies found that BIs conducted among patients attending emergency care did not result in significant reductions in alcohol intake but did result in diminished odds of alcohol-related injuries [27]. While study heterogeneity in terms of design, selection of screeners, and outcome measures may account for these findings, more research is needed before conclusions about the general effectiveness of BI in emergency room settings can be drawn [28]. 

Third, most studies of SBI for AOD use within emergency departments originate from high income countries and little is known about the feasibility or effectiveness of SBI for AOD problems in low and middle income country (LMIC) contexts, such as South Africa. This is cause for concern as studies conducted in high-income countries report culture- and country-specific barriers that affect patients' responses to SBI and the process of implementing SBI in emergency care [28, 29]. Although studies conducted within South African general health services suggest that there are likely to be barriers to SBI implementation that include physician reluctance to raise questions about AOD use (due to concerns about patient responses to the question) [30], little is known about barriers to implementation within the context of emergency care and how these barriers impact SBI outcomes. These limitations are problematic as they potentially impede the development of culturally appropriate interventions to reduce AOD use among patients attending emergency rooms. 

Finally, there has been little research on whether SBI programs for AOD problems conducted in emergency care settings are feasible to implement in countries such as South Africa where health care resources are limited [5]. In high-income countries (which have relatively well-resourced health systems) nurses and physicians are generally responsible for delivering SBI [28]; however, LMICs often have chronic shortages of health personnel and are unlikely to support SBI programs that increase the work burden of scarce health professionals [31]. Task-shifting SBI from health professionals to peer counselors has been proposed as one strategy for overcoming these resource limitations in LMICs [31]. While peer-led AOD interventions have been successfully implemented in community settings [32], there are few published studies examining the effectiveness of peer-delivered SBI programs for AOD use within emergency rooms. 

This paper begins to address these gaps by reporting findings from a process evaluation of an on-going peer-led screening and brief motivational intervention program for AOD problems (Project STRIVE) conducted in emergency room settings in Cape Town (the capital city of the Western Cape), South Africa. More specifically, this paper aims to describe (i) the feasibility of screening and conducting brief interventions for AOD problems among patients presenting with injuries at emergency room settings in Cape Town, (ii) patients' and emergency room personnel's responses to the peer counselor-led screening and brief AOD intervention program, and (iii) peer counselors' perceptions of barriers and facilitators to conducting SBI for AOD use within emergency room settings.

## 2. Method and Materials

### 2.1. Study Sites

This on-going pilot program is being implemented at three 24-hour emergency room services in two large impoverished communities in the Cape Town metropole. These public emergency room services were purposively selected by the Western Cape Department of Health as sites for the program because of the high proportions of alcohol-related homicides in these areas and the large numbers of patients treated for AOD-related injuries [6]. Patients presenting for these emergency room services are first triaged by a nurse into one of the following categories of problem severity: (1) red comprising patients who are physiologically unstable and may require resuscitation; (2) orange consisting of serious cases with potentially unstable physiology or threatening pathology; (3) yellow consisting of physiologically stable patients; and (4) green comprising patients with minor injuries or illnesses [33]. Due to the high trauma case load seen at these services and the shortage of nurses and doctors in emergency services [34], patients in the green and yellow triage categories often wait for several hours before being attended to. 

### 2.2. Program Description

At each selected study site, peer counsellors approach patients for screening after they have been triaged and while they wait to be seen by the attending doctor. The triage nurses also refer patients who they think may be suitable candidates for the program to be screened. Patients are screened and recruited during varying times during the day and during at least one 12-hour night shift on the weekend (7pm–7am) reflecting the busiest periods of the selected emergency services. Due to the pilot nature of this intervention program and the triage system within emergency services it is not possible to screen all patients who present for care. Therefore, the counselors do not approach those patients with overt exclusions (such as those younger than 18 years of age or mothers seeking care for their children) for screening.

More specifically, counselors approach patients as they wait for care by introducing the intervention program and providing an overview of the goals of the program and potential benefits to the patient. Counselors also explain to potential participants that screening positive for AOD use will not jeopardize their access to health services or compromise the quality of care received. Patients are then asked to provide consent to be screened for eligibility to participate in the intervention program. 

 To be eligible for the intervention program, participants must be at least 18 years of age, present with an injury, and screened at moderate or high risk for AOD-related problems using the Alcohol, Smoking, and Substance Involvement Screening Test (ASSIST, [35]). The ASSIST was originally developed to detect and manage AOD use in primary and general medical care settings and as such categorizes people into low, moderate, or high risk for AOD-related problems. Low risk indicates that the participant is at low risk for health and other problems from their current pattern of AOD use (with scores of 0–10 for alcohol and 0–3 for illicit drugs). Moderate risk indicates that the person is at risk for health and other problems from their current pattern of AOD use, with scores of 11–26 for alcohol and 4–26 for illicit drugs. Scores >26 indicate that the participant is at high risk of experiencing severe problems as a result of their current pattern of use and is likely to be dependent [35].

As low risk users are not eligible for the intervention, they are thanked for their time and encouraged to maintain low risk usage. Eligible moderate and high risk users are asked for consent to participate in an AOD risk reduction program. Those patients who consent then complete an interviewer-administered baseline questionnaire. This questionnaire covers item domains pertaining to AOD use, injury as well as other health risks (such as depression and interpersonal violence) and takes approximately 45 minutes to be completed. Immediately after the baseline assessment, participants are provided with a peer counselor-delivered ASSIST-linked brief motivational intervention [25].

This manual-guided brief intervention is a short, one-session, intervention delivered in an individual format during which time the peer counselor (i) raises the subject of AOD use; (ii) provides structured feedback about the risks associated with their current pattern of AOD use by reviewing the patient's ASSIST scores for all substances used (and not just their primary drug of choice) and where possible connecting their AOD use to their injury; (iii) gives the patient concrete advice about the need to change his/her current pattern of use to reduce their health and injury risks; and (iv) enhances motivation and readiness to change through the use of motivational interviewing techniques such as the use of reflective listening, open-ended questions, eliciting change talk, and affirming positive steps to change [25]. The duration of this brief intervention is approximately 30 minutes and the intervention is conducted in a private room within the emergency services. In addition to this brief intervention, patients who screen at high risk for AOD-related problems are referred to specialist AOD treatment facilities for further treatment. 

All of the peer counselors who conducted the brief intervention have a bachelors-level education and originate from the communities served by the selected emergency services. These peer counselors completed a 3-day training in motivational interviewing (with proficiency testing through role-playing and case examples) provided by a motivational interviewing-certified trainer and also received 3 half-day booster trainings to limit intervention drift and ensure that the motivational interviewing skills were being applied appropriately. In addition to the intervention training, peer counsellors received 16 hours of training in (i) alcohol and illicit drugs and the risks associated with AOD use, (ii) using and scoring the ASSIST, (iii) ethics, (iv) the intervention program procedures, and (v) the process of referring patients for specialized care. To ensure intervention fidelity, peer counselors are required to participate in biweekly supervision and debriefing sessions. 

Participants are followed up 3 months subsequent to the intervention during which time the baseline questionnaire is readministered and a feedback questionnaire about the services is completed. In order to limit a socially desirable response set, the follow-up assessment and feedback questionnaire are not administered by the peer counsellor who delivered the initial intervention. As this program was developed with the intention of being integrated into existing health services, participants receive an incentive for participating in the initial baseline assessment and in the evaluation of the program. Specifically, participants are given a grocery store voucher valued at ZAR 30 (about USD 4) for completing the baseline measures and after completion of the final assessment. 

At no point this screening and brief intervention program is allowed to interrupt patient flow in emergency services. Therefore, if the patient is called to see the emergency doctor while being screened or during the course of the intervention, the counselor halts the intervention and makes arrangements with the patient to resume the program at a later point in time after the patient has received emergency care. 

### 2.3. Process Evaluation Design and Procedures

This process evaluation employed a mixed methods design comprising four components: (i) a quantitative part that examined patient throughput to assess the feasibility of screening and recruiting patients for an emergency room intervention, quantitative aspects that assessed (ii) patients' and (iii) emergency personnel's preliminary responses to the intervention, and (iv) a qualitative aspect that examined peer counselors' perceptions of barriers and facilitators to deliverg and scale up AOD interventions in emergency room settings.

For the first part, data were extracted from program records on the number of patients screened, the number of eligible patients relative to the total number of patients screened, and the number of patients who agreed to participate in the program relative to the number of eligible patients. For patients who refused to participate in the program, reasons for not wanting to participate were recorded. 

For the second part of the evaluation, a semistructured intervention feedback questionnaire was used to examine preliminary responses to the intervention. While this questionnaire is administered to all patients after they complete their three-month follow-up interview, here we report only on findings from the first 30 patients. The questionnaire included items pertaining to the usefulness and impact of the program for identifying problematic AOD use patterns and reducing AOD use. Patients also were asked about their willingness to attend additional intervention sessions as an indicator of the acceptability of the intervention. 

Similarly, for the third part of the evaluation, a semi-structured questionnaire was administered to emergency personnel from each site to examine their preliminary responses to the SBI program. Fifteen emergency room personnel were approached to participate in these interviews. Of the 15 personnel approached, three were not aware of the services offered and therefore declined to participate in the interviews and two declined to participate due to time constraints. The questionnaire that was administered to the remaining 10 emergency room personnel included items pertaining to their perceptions about the usefulness of the peer counselors and SBI program, areas in which peer counselors could have improved, factors that would have encouraged emergency personnel to refer patients to the SBI program more frequently, and perceptions about whether the program helped or hindered services within the emergency department. 

For the final aspect of the evaluation, an open-ended semi-structured interview schedule was used to elicit possible factors that hindered or supported the implementation and execution of the screening and brief AOD intervention program within the context of emergency services. All five peer counselors, responsible for the delivery of the screening and intervention program, were interviewed by an experienced interviewer. These in-depth interviews were audiotaped and transcribed verbatim before the textual data were analysed using qualitative techniques. 

Specifically, the qualitative data analysis for this study was conducted using the framework approach (familiarization, identifying a thematic framework, indexing, charting, mapping, and interpretation of the data). Initially, interview transcriptions were read for emergent themes, which were then coded. Care was taken to ensure the codes accurately captured the respondent's meaning. A second researcher independently coded the interviews to ensure validity of the categories. We used NVivo 9.0, a qualitative software program for data analysis. 

Ethics approval for the evaluation was provided by the Research Ethics Committee from the University of Cape Town's Faculty of Health Sciences.

## 3. Results

### 3.1. The Feasibility of Screening and Recruiting Patients for an Emergency Room Intervention

In the first three months of the program, a total of 1458 patients presenting for emergency services were screened for possible AOD use. Of these patients, 270 (18.5%) were screened during the early shift (7am to 1pm), 650 (44.5%) during the afternoon shift (1pm to 7pm), and 538 (36.8%) during the night shift (7pm to 7am). Patients at South African emergency rooms are triaged according to injury severity and in most instances patients with severe injuries requiring immediate medical intervention were not able to be screened. Of the patients who were screened, 20.9% (*N* = 305) were considered at moderate to high risk for an AOD use disorder thus meeting criteria for participation in the intervention study. Of these 305 patients, 225 (73.8%) participants were willing to participate in the intervention program. Among the 83 eligible patients who refused the intervention, the two most frequently reported reasons for not wanting to participate were that they were experiencing too much pain and that they felt they did not have an AOD problem. Although the 83 participants who qualified for but refused the intervention had significantly lower scores on the ASSIST (*M* = 15.9, SD = 9.4) than the 225 participants who qualified for and were willing to participate in the intervention (*M* = 18.2, SD = 7.4; *t* = −2.34, *P* = 0.02), the mean ASSIST scores of both groups were both within the “moderate risk” category.

### 3.2. Patients' Preliminary Responses to the SBI Program

Of the 30 patients who have participated in the three-month follow-up interview to date, the majority were women (*n* = 18; 60%), with an average age of 29 years old (SD = 13). Most participants were single (*n* = 22; 73.3%), unemployed (*n* = 22; 73.3%) and did not complete high school (*n* = 20; 66.7%). Fourteen (46.7%) of these patients presented themselves to the emergency department as a result of a violent assault, and 18 (60%) reported that they were under the influence of AODs when they presented at the emergency services. Twenty-six of the 30 participants (86.7%) reported alcohol as their primary substance of abuse, 3 (10.0%) reported problems associated with cannabis use, and 1 (0.03%) reported problems associated with methamphetamine. 

When asked for feedback about the screening and intervention program, 22 (73.3%) of the 30 participants felt that the screening tool was useful in helping them to understand the level of risk associated with their current pattern of AOD use and that the educational information provided during the structured feedback helped them understand the positive and negatives of using their drug of choice. Regarding whether or not the intervention provided actually helped participants cut down or stop using AODs, only 1 participant (3.3%) felt it was “not useful,” 13.3% (*n* = 4) thought it was “somewhat useful” and the majority (*n* = 25, 83.3%) reported it was “useful.” In addition, 43.3% (*n* = 13) of participants wished that they could have had more than one session with the counselor in order to further address their AOD use. 

When these 30 participants were asked about their willingness to attend additional counseling sessions, 25 participants (83.3%) reported that they would be willing to return to the clinic for one, 20 participants (66.7%) for two, 14 (46.7%) for three, and 11 (36.7)% for four additional counseling sessions.

### 3.3. Emergency Room Personnel's Preliminary Responses to the SBI Program

Of the 10 emergency room personnel who participated in this aspect of the evaluation, most (*n* = 8) were female and were on average 34 years old (range = 26–53 years). While the vast majority had nursing qualifications (*n* = 8), two participants responsible for the management of these emergency room services were also interviewed. 

While only five of the ten respondents had referred patients for screening who they thought might benefit from the SBI program, all of the respondents felt that the SBI program was useful and that the peer counselors were helping trauma patients with AOD problems. When asked for examples of how the program was useful to and valued by patients, several respondents described how some patients returned to the emergency room looking for the peer counselors after completion of the SBI program. In addition, all of the respondents felt that the SBI program did not interfere with their workflow in the emergency room, nor did the presence of the peer counselors and the SBI program add to their workload. 

Although all of the respondents were in favor of the continuation of this pilot SBI program for AOD use, they did identify areas in which the SBI program could be enhanced. Six of the ten respondents recommended expanding the reach of the program. Specifically they felt that the program should be expanded to enable 24-hour availability of a peer counselor at the emergency room so that all patients who could potentially benefit from SBI for AOD use were able to access this service. In addition, two of the ten respondents recommended improving the visibility of the program and peer counselors to emergency personnel and also to patients as a means of encouraging the uptake of these services. Specifically these respondents suggested that the marketing of the program could be improved through the use of posters and program fliers. Finally two of the ten respondents recommended expanding the content of the BI to address other major psychosocial issues that intersect with AOD use (such as depression and gender-based violence) and affect patients who present with injuries at emergency rooms.

### 3.4. Peer Counselors' Perceptions of Barriers and Facilitators to Deliver SBIs for AOD Use in Emergency Rooms

Peer counselors identified several factors that need to be considered when scaling up the implementation of AOD interventions in emergency room settings. These relate mainly to barriers within the structural and cultural context of the emergency room setting, patient-level barriers, and counselor-level factors. 

#### 3.4.1. Barriers within the Structural and Cultural Context of the Emergency Room Setting

All of the counselors felt that the emergency room personnel were not adequately informed about the program by the health facility's management team. Three of the five respondents felt that they had to explain the program and its objectives numerous times to ensure that emergency room personnel were aware of the services being offered to patients. One counselor gave an example of this poor communication:

* “On my first day when the management was here, everything was good, but when they left, then everything started to be a little bit difficult—because the staff didn't understand the purpose why we're here as the counselors. We had to introduce ourselves over and ourselves over and over. But after a few days we started getting used to the system they started understanding why we're here. But still there were a little bit of difficulties.”*

Although counselors reported that most emergency room personnel expressed the importance of an AOD intervention for at-risk patients, support and buy-in for the program differed between the intervention sites. Following the first two months of implementing the intervention, two of the three sites reported that almost all emergency room personnel were open to worked with the counselors to identify and refer at-risk patients. However at one of the sites, few personnel were open to collaborate with counselors to ensure the success of the program. The following comments reflect this poor support for the program:

*“You made it sound very nice and organised and all of that, you know, in the beginning—but when we got here it's not at all like that. It's still not like that. You know, we still have to go into trauma and say again who we are, what we're doing here. So far there's only been two sisters ever since we started that sent people our way; the others just do not. I do not know what it is but they just do not.”*

*“We do have problems with referrals from the trauma room to us. We have to walk around and approach people. There was confusion because the doctors, patients, trauma patients, dentist patients, are sitting at the same spot—so it's difficult to differentiate between who's who. And, ja, but overall I won't say it was a nice part, but it has ups and downs; and as a person I have grown because I had to like problem solve, every day had its own problems, and then you had to deal with it as it came.”*

A third structural difficulty was the lack of private space within the emergency room where brief interventions could be delivered in a confidential manner. This was a major hurdle to the delivery of the intervention in two of the three sites. Several attempts were made to find a permanent solution to this challenge, but clinic priorities and a general lack of space in the facilities made this an impossible problem to permanently solve within the current parameters of these emergency room services. This was encapsulated in the words of one counselor:

*“The first month was hectic because we did not have office space. I think it was the second month where we started to have some space and eased into the process; a process that I do not think is established as such.”*

#### 3.4.2. Patient-Level Barriers

The main patient-level barrier to delivering the screening and intervention program relates to the characteristics of the patients presenting for emergency services. All of the counselors reported concerns about approaching patients who were severely injured, very intoxicated, or had been waiting a long time to be seen by emergency personnel for screening and possible participation in the intervention. These concerns were particularly salient during weekend shifts when emergency services were inundated with aggressive and intoxicated patients. To illustrate, one counselor noted that

*“It's just so hard to even approach a person who is so aggressive and violent that he is yelling at everyone he sees, demands to be seen yesterday, and looks like he was just in a gang fight and probably is still drunk or still on tik.”*

Patient reluctance to disclose AOD use was another patient-level barrier to delivering the program as planned. Although in some cases, the counselors felt that the patients were comfortable disclosing their AOD use, there were times where it was obvious that the patient was withholding information. Some patients also struggled to understand why they were being asked about their AOD use when they were presenting with an injury that required medical attention. This is the conveyed through the following statement:

* “We had quite a few youngsters and female girls—you can see they are, you know, you can smell that they've been drinking and they will say they've been drinking the whole Friday and Saturday—and now they come whatever the incident is. And then you ask, ‘Were you drinking at the time?' and they say ‘No.' But I don't know if they think you're going to charge them or you're with the cops or something.”*

#### 3.4.3. Counselor-Level Factors

An important counselor-level factor that needs to be considered when scaling up this intervention program is the personal safety of counselors. Three of the five counselors expressed that they were apprehensive about their personal safety (due to working with perpetrators of violent acts) during training for the project. However these concerns about safety seem dependent on the length of time working in the emergency units. Five months after the program was initiated, none of the counselors reported being overly concerned about their safety within the health facilities due to the strict regulations employed to ensure staff safety. This is highlighted below:

* “The safety, I will never complain, I never say anything about the safety, like being in danger. I always feel safe because there are securities at the gate and there's always a security next to the doors there. The security station is right across from our office that we are using.”*

However some counselors were still concerned about their safety outside of these facilities, which were located in high-crime areas. Two of the counselors expressed concern over travelling to and from the emergency unit, particularly for their overnight shifts. As one counselor stated

* “Travelling sometimes is not that safe, especially if you're using public transport because you need to take a taxi at around about half six, but at summer it was fine because it was not dark. But now it's winter, it's a little bit dark to walk over the bridge and come to the hospital. But in the hospital it's quite safe.”*

A second counselor-level factor that needs to be considered when scaling up this intervention program is the adequacy of training for peer counselors in terms of equipping them to cope with injured patients. Specifically, all the counselors felt they were not completely equipped (even after extensive training) to deal with the traumatic nature of working in an emergency room setting. Over time, the corrective measures taken to address this (such as biweekly supervision and debriefing meetings) and peer support helped them feel more comfortable dealing with the patients that present for emergency services. This is illustrated below:

*“…now I think I'm getting used to it. At first I felt traumatised by seeing such volumes of blood. So that was a shock. Yes. But now I think I'm used to it. Especially on weekends when we do our nightshifts on weekends—the first nightshift on weekend—you see that the floor is white—it was red covered with blood and you could even smell the blood.”*

When asked about the appropriateness and duration of the training, all counselors felt that the training (in conjunction with booster training, role plays and rehearsal opportunities provided to counselors) was more than sufficient to teach them the basic principles of motivational interviewing. They also felt that the skills and information they gained through this intensive training helped them deal with issues in their everyday lives. The following comments illustrate these personal benefits:

*“To be honest, the training has it benefited me in different ways. Starting from the first day that I worked as a counselor here in Khayelitsha, even I've changed my own behavior, so it has helped me a lot as a person. I'm no longer walking out at night, at seven o'clock I'm indoors. At home everything just changed, I'm relating too much to my family—so everything is easy for me than it was before—understanding the different problems that people face, so I tend to take mine as minor problems.”*

*“I think personally I have grown since I've started doing this. You see people opening up, and you see people opening up even in areas that you never thought that they would be willing to open up to a stranger, but they do. And, you know, each client, each case is different from the other. And when you sit back and review in your mind whatever session you had, there's an experience to take with you and grow and learn, so I think I'm growing.”*

## 4. Discussion 

This paper is among the first to examine the feasibility and acceptability of conducting SBI for AOD use within emergency room settings in an LMIC context. More specifically, findings from this study show that, with the addition of minimal resources, such interventions are feasible to conduct in LMICs among populations with low levels of formal education and high levels of unemployment, are acceptable to patients, and have promising outcomes. 

First, findings indicate that it is not only feasible to screen large numbers of people presenting for emergency services in Cape Town, South Africa, for possible inclusion in an AOD risk reduction intervention, but that such an intervention program is needed among this population. More specifically, the need for an AOD risk reduction intervention was high among the patients screened, with over a fifth of patients screened meeting criteria for moderate to high risk AOD use. Nonetheless, this is probably an underestimation of the magnitude of the AOD intervention need since the peer counselors were reluctant to approach overtly intoxicated patients who were acting aggressively and because some patients were hesitant to disclose their AOD use. In addition, counselors were unable to screen patients with severe injuries requiring immediate medical intervention for possible inclusion in the program. It is likely that these patients were disproportionately affected by AOD use, especially as earlier studies reported positive associations between AOD use and risk of serious injury [4–8]. Despite this, our findings clearly show that screening for AOD use in emergency services in Cape Town yields a significant number of patients who could benefit from an AOD risk reduction intervention. In addition, we found that a high proportion (close to three-quarters) of these eligible patients were willing to participate in a brief intervention to reduce their AOD use, thus demonstrating the feasibility of recruiting patients from emergency room settings to participate in brief AOD interventions. Taken together, these findings demonstrate that it is feasible to conduct SBI for AOD use within emergency rooms in Cape Town, South Africa. 

Second, our findings demonstrate that a brief motivational intervention to reduce AOD use has high levels of acceptability and utility to patients presenting for emergency services in Cape Town, South Africa. The vast majority of patients who provided feedback about the intervention reported that the intervention was useful for helping them understand the risks associated with their AOD use and for helping them reduce their AOD use. Further evidence of the acceptability of this intervention is that the vast majority of patients interviewed were willing to return to the health facility to attend at least one additional counseling session and expressed a desire for additional counseling sessions. Emergency room personnel also noted that patients seemed to value the program. The high levels of acceptability and perceived utility of this brief motivational intervention suggest that it would be worthwhile testing the effectiveness of this intervention in a future randomised controlled trial.

Third, our findings demonstrate that SBI for AOD use can be implemented in emergency room settings in an LMIC with low levels of investment in additional health resources and with little disruption to health care delivery. Specifically, this SBI program was entirely peer counselor driven and therefore required little additional investment in costly (and scarce) health care personnel. It also did not require existing emergency room personnel to take on additional responsibilities of screening for AOD use. In fact, one of the aspects of the program that emergency personnel liked was that it did not add to their existing workload. According to emergency personnel, peer counselors were also able to blend into existing emergency services and provide SBI without disrupting patient or work flow. Taken together, these findings suggest that task-shifting responsibility for SBIs from emergency room personnel to peer counselors may be a viable solution to some of the commonly cited barriers to implementing SBIs for AOD use in emergency room settings, particularly thehigh workload of health care professionals, and limited resources for implementing additional AOD intervention services in emergency care [28].

While our findings provide preliminary evidence that such a peer-led intervention holds promise for facilitating changes in AOD use, we learned some valuable lessons that need to be considered when scaling up the implementation of AOD interventions and research in emergency room settings. First, more effort needs to be taken to ensure that peer counselors are fully integrated into the emergency room team. Better integration of peer counselors into emergency services would potentially address many of the structural and contextual barriers (such as poor communication and lack of referrals from emergency personnel) that may have negatively impacted on the implementation of the intervention. Second, we learned that peer counselors need extensive training and on-going support (via debriefing and regular supervision) to help them cope with patients who are perceived to be dangerous and with working in emergency room settings which are often traumatic for people without health training. Future efforts to scale up this intervention therefore would benefit from providing peer counselors with on-going support and supervision to help them cope with working in this challenging environment. While the provision of additional training and support to peer counselors will have cost implications for the programme, a peer counselor-driven AOD intervention is still likely to be less expensive to implement than a program delivered by health professionals. To substantiate this claim, cost-effectiveness studies that compare the cost benefits of AOD interventions delivered by peer counselors and health professionals are required. 

While both patients and emergency room personnel thought that this pilot SBI program was useful and should be continued, findings from this study should be considered in the light of several limitations. First, given the pilot nature of the program, we were unable to conduct universal screening within the selected emergency rooms and therefore could not assess the prevalence of problematic AOD use within these settings. Future studies should consider implementing universal screening so that all patients who may potentially benefit from SBI for AOD use are able to access this service. Secondly, we did not record the number of patients present in the emergency room during screening hours and therefore were unable to assess the reach of the SBI. Although assessing reach or service coverage was beyond the scope of this small feasibility study, future studies should collect this information so that the potential impact of this SBI program can be properly evaluated. Third, we did not record the number of patients who were approached but refused to be screened and as a result we were unable to assess the degree to which screening for AOD use within emergency care was acceptable to patients. Finally, findings about the patient responses to the intervention should be interpreted with caution given the very small sample size and the lack of a control group. 

## 5. Conclusion

Despite some limitations, results from this study suggest that it is feasible to conduct SBIs to reduce AOD use among patients presenting for emergency services in an LMIC such as South Africa with minimal additional health resources. Although larger studies are needed to test the effectiveness of SBI for reducing AOD use and preventing future AOD-related injuries, findings from this study suggest that a peer-led SBI program for AOD use is feasible to implement in emergency care and is acceptable to both patients and emergency service providers. Findings also provide valuable insight into how best to address potential barriers to the implementation of SBIs at the process, counselor and patient levels, thereby increasing the likelihood of an effective randomized controlled trial. 
